# Analysis of localized immune responses reveals presence of Th17 and Treg cells in cutaneous leishmaniasis due to Leishmania tropica

**DOI:** 10.1186/1471-2172-14-52

**Published:** 2013-11-22

**Authors:** Gajendra Kumar Katara, Anand Raj, Rajesh Kumar, Kumar Avishek, Himanshu Kaushal, Nasim Akhtar Ansari, Ram Awatar Bumb, Poonam Salotra

**Affiliations:** 1 National Institute of Pathology (ICMR), Safdarjung Hospital Campus, New Delhi 110029, India; 2 Department of Dermatology, STD and Leprosy, SP Medical College, Bikaner, Rajasthan, India; 3 Present address: Department of Microbiology & Immunology, Rosalind Franklin University of Medicine and Science, North Chicago, IL 60064, USA; 4 Present address: Laboratory of Erythropoietin, National Dope Testing laboratory (NDTL), JN Stadium Complex, New Delhi 110003, India; 5 Present address: Department of Tropical Medicine, Tulane University, School of Public Health, New Orleans, LA 70112, USA; 6 Present address: Medical Research Centre, Jazan University, Jazan, Saudi Arabia

**Keywords:** cDNA array, Cutaneous Leishmaniasis, Cytokines, Th17 cell, Treg cell

## Abstract

**Purpose:**

The interaction between the *Leishmania* parasite and the host cell involves complex, multifaceted processes. The disease severity in cutaneous leishmaniasis (CL) is largely dependent on the causative species. Most of the information on immune responses in human CL is available with respect to *L. major* infection and is lacking for *L. tropica* species. In this study, we employed cytokine/chemokine/receptor membrane cDNA array to capture comprehensive picture of immuno-determinants in localized human tissue during *L. tropica* infection. Expression of selected molecules was evaluated by real time PCR in dermal lesion tissues at pre- and post treatment stages. Plasma IL-17 level was estimated by sandwich ELISA.

**Results:**

The cDNA array analysis identified several immuno-determinants in tissue lesions of Indian CL including cytokines (IFN-γ, TNF-α, IL-1β, IL-10, IL-13), chemokines (IL-8, CCL2, CCL3, CCL4) and apoptotic molecules (Fas, TRAIL, IRF-1). Elevated mRNA levels of Th17 (IL-17, IL-23 and RORγt) and Treg (CD25, CTLA-4 and Foxp3) markers were observed in lesion tissues of CL patients compared to the control group, which subsided post treatment. Plasma IL-17 levels were found to be significantly higher in CL samples compared to controls.

**Conclusions:**

In addition to defining comprehensive immunological responses inside lesion tissues of CL patients, our study demonstrated the presence of Th17 and Treg cells in CL caused by *L. tropica*.

## Background

Leishmaniasis represents a group of neglected infectious tropical diseases caused by obligatory intracellular, protozoan parasites belonging to the genus *Leishmania*. The infection results in a variety of clinical syndromes ranging from benign skin lesions to terribly disfiguring mucosal lesions, and the potentially fatal visceralizing form. The disease is currently endemic in 98 countries or territories and overall prevalence is estimated as 12 million with 350 million at risk [1]. Cutaneous Leishmaniasis (CL) is the most common form of leishmaniasis with the worldwide annual incidence as 1–1.5 million. More than 90% of CL cases occur in 10 countries: Afghanistan, Algeria, Brazil, Bolivia, Colombia, Iran, Nicaragua, Peru, Saudi Arabia and Syria [1]. In India, CL is endemic in Western Thar region of Rajasthan particularly in Bikaner region with *L. tropica* as the major causative agent [2]. The clinical manifestation varies from spontaneous healing lesions to chronic mutilating and diffuse cutaneous lesions [3]. Moreover, it is fraught with problems, such as high toxicity or drug resistance particularly in case of CL-HIV co-infection [4,5].

Published data using animal models or humans for leishmaniasis caused by *L. tropica* is limited, in part because of difficulties in establishing infection *in vivo* and other practical difficulties [6]. Studies from our laboratory have documented significant elevated mRNA levels of interferon (IFN)-γ, tumour necrosis factor (TNF)-α, monocyte chemoattractant protein (MCP)-1, interleukin (IL)-10, IL-1 β, IL-8 and IL-4 in localized lesion tissues of CL patients [7]. In addition, we also reported correlation between IL-4 with parasite load in early lesions tissues of CL [8]. In spite of the presence of effector molecules such as IFN-γ, TNF-α and NO during active disease, the parasite persists, implying that the biological processes involved in the disease pathogenesis are complex and cannot be interpreted as simple T helper 1 (Th1) or Th2-mediated processes, characteristics of the murine model of leishmaniasis.

In recent times, the role of Th17 and Treg cells has been implicated in human leishmaniasis. IL-17 producing CD4^+^ T cells (Th17) have recently been defined as a separate T cell lineage, which plays an important role in defense mechanisms against certain pathogens [9]. In human visceral leishmaniasis (VL), IL-17 is associated with protection whereas in human CL, it is associated with infiltration and disease pathobiology [10,11]. Similarly, another subpopulation of CD4^+^ T cell, which is CD4^+^CD25^+^Foxp3^+^ regulatory T (Treg) cells are unique for their ability to inhibit the response of other T cells. Evidence from experimental murine models of *L. major* infection suggests that nTreg cells promote survival of *Leishmania* parasites and reactivation of disease [12]. In addition, in human CL intralesional Treg is associated with SAG unresponsiveness and disease pathology [13,14].

Despite substantial progress in *Leishmania* immunobiology, studies focusing on immunological profile of patients with *L. tropica* infection are few and correlates of protective immunity are ill defined. In this study, we utilized cDNA array technology to obtain an *ex vivo* comprehensive immunological scenario inside lesion tissues of CL patients, demonstrating the presence of Th17 and Tregs cells in CL pathogenesis.

## Methods

### Tissue and blood samples

Punch biopsies (4 mm) were collected from skin lesions of CL patients reporting to the Department of Skin, STD & Leprosy, S. P. Medical College, Bikaner (Rajasthan), India. Active CL was diagnosed clinically by the appearance of lesions, present on exposed area of the body and was confirmed by microscopy and/or PCR [7]. The biopsies were collected 3–4 days prior to start of treatment in RNAlater (Ambion, Austin, TX, USA) and stored in liquid nitrogen until use. Total RNA was isolated using Trizol reagent, in accordance with manufacturer’s instructions, and quality of RNA was assessed using Bioanalyzer (Agilent, Foster City, CA, USA). Blood samples were collected in heparin-anticoagulant vacutainer tubes, plasma isolated by centrifugation and stored at −80°C until use. Sixteen confirmed cases of CL in the age range of 15–51 years were included in this study, among them 10 (62.5%) were male and 6 (37.5%) were female. The clinical history ranged from 1–24 months (Table 1).

**Table 1:** Clinical features of the study population

**Patient characteristics**	**CL (n = 16)**
**Age (years) range, (mean ± SD)**	15-51, (24.37 ± 10.05)
**Sex (M/F)**	10/6
**Duration of CL, range in months, (mean ± SD)**	1-24, (5.90 ± 6.55)
**Types of lesions**	
Erythematous Ulcerated Plaque	9
Erythematous Ulcerated Nodule	5
Ill defined plaque	2

*Abbreviations: M*, Male; *F*, Female; *CL*, Cutaneous leishmaniasis.

Patients were given treatment with sodium antimony gluconate (SAG) intralesionally, 0.5 ml/cm^2^ of lesion, twice a week for 5–7 injections, depending on the response to treatment. Alternatively, in cases with multiple lesions, rifampicin (20 mg/kg body weight) was given for 3 months orally. Follow up samples (n = 7) were collected from the same site as at pre treatment stage, 2–4 weeks after completion of treatment and apparent clinical cure. Normal skin biopsy samples (n = 7) were collected as controls from healthy volunteers from endemic area. The study was approved by and carried out under the guidelines of the Ethical Committee of the S.P. Medical College, Bikaner, India. Informed consent was obtained from all patients or their guardian.

### Analysis of mRNA expression using cDNA arrays

Total RNA was isolated from punch biopsy samples from CL patients (n = 6) and healthy individuals (n = 6) using Trizol (Invitrogen, Green Island, NY) method. RNA samples were pooled in equal amount from 6 individuals in each group. Ten micrograms of DNA-free RNA from each group was reverse transcribed, in the presence of 50 μCi of α-^33^P dATP (specific activity ≥ 2000 Ci/mmol; (Perkin Elmer, San Jose, CA) and gene specific primers for each gene represented on the array. The cDNA microarray (AtlasTM; CLONTECH, Palo Alto, CA) consisted of nylon membranes, spotted with 268 different human genes including those encoding cytokines, chemokines, growth factors, and cellular receptors (http://www.clontech.com/support/tools.asp). Briefly, [^33^P] dATP-labelled cDNA was column purified and hybridized, at high stringency, to cDNA array overnight at 68°C. Membranes were washed at high stringency and exposed to phosphor screens for overnight. Image was captured with phosphorImager Typhoon 9210 and analyzed by Imagequant TL software (Amersham Biosciences, Pittsburgh, PA). The intensity of each spot was corrected for background levels and normalized using the housekeeping genes. Array data was analyzed by summing the duplicated intensity signals for each gene in individual experiments and taking the average of 3 technical replicates. The data was expressed as the ratio of mRNA levels in CL and controls.

### Quantitative mRNA analysis

Real-time PCR was performed as described earlier [15], using gene specific FAM-MGB–labeled Taqman primer sets (Applied Biosystems, Foster City, CA) for IFN-γ (Hs00174143_m1), IL-10 (Hs00174086_m1), MCP-1 (Hs00234140_m1), IRF-1 (Hs00971965_m1), Fas (Hs00384673_m1), IL-1β (Hs01555410_m1), IL-17 (Hs00174086_m1), IL-23 (Hs00166229_m1), RORγt (Hs00175480_m1), CD25 (Hs00166229_m1), CTLA-4 (Hs00175480_m1) and Foxp3 (Hs00203958_m1). FAM-MGB β-actin (Hs99999903_m1) was used as endogenous control. The relative quantification of products was determined by the number of cycles over endogenous control required to detect the gene expression of interest.

### Detection of IL-17 in plasma

IL-17 levels in plasma of CL patients (n = 15) and controls (n = 10) were determined by sandwich ELISA (e-Biosciences, San Diego, CA) in accordance with manufacturer’s instructions. Each sample was tested in duplicate, and cytokine concentrations were calculated using a standard curve generated from recombinant cytokines. Cytokine values were expressed as picograms per milliliter.

### Statistical analysis

Statistical analysis was performed with Mann–Whitney test/paired t-test using Graph Pad Prism 5 (GraphPad Software, Inc., San Diego, CA). *P* values ≤ 0.05 were considered significant.

## Results

### Gene expression profile in CL

cDNA array was used to capture gene expression profile in lesion tissues of CL patients. Seventy one genes out of 268 arrayed genes (26.4%) which included cytokines, chemokines, receptors and other regulatory molecules, showed modulation of 2 fold or more in cutaneous lesions tissue compared to control. Table 2 lists selected 46 genes showing altered expression during CL.

**Table 2:** Genes showing altered (>2 fold) gene expression in tissue lesions of CL compared to human normal skin (Control)

**Gene name**	**Gene accession no.**	**Major function**	**Relative mRNA expression (CL/Control)**
**Cytokines & Chemokines**			
Interleukin (IL)-8	Y00787	Chemo-attractant and inflammatory response	22.51
Interferon (IFN)-γ	X01992	Immunoregulatory and anti-tumor properties, activator of macrophages	15.32
IL-12β	M65290	Inducer of IFN-γ, differentiation of both Th1 and Th2 cells	14.87
Monokine induced by gamma interferon (CXCL9)	X72755	T cell trafficking	12.47
Tumor necrosis factor (TNF)-α	X01394	Proinflammatory cytokine, cell proliferation, differentiation, apoptosis	10.81
C-Chemokine ligand (CCL4) or (MIP-1β)	J04130	Chemo-attractant of monocytes/macrophages	9.65
Granulocyte macrophage colony stimulating factor (GMCSF)	M11220	Production, differentiation, and function of granulocytes and macrophage	6.16
IL-13	L06801	Inhibition of production of pro-inflammatory cytokines and chemokines	5.50
CCL-3 or (MIP-1α)	M23452	Inflammatory responses	4.38
Transforming growth factor (TGF)-α	K03222	Cell proliferation, differentiation and development	4.15
IL-10	M57627	Pleiotropic effects in immunoregulation and inflammation	3.92
CXCL-5	X78686	Inflammatory chemokine involved in neutrophil activation	3.81
IL-17	U32659	Proinflammatory cytokine, high levels are associated with several chronic inflammatory diseases	3.77
IL-1β	K02770	Mediator of the inflammatory response, involved in cell proliferation, differentiation, and apoptosis	3.75
IL-6	X04602	Functions in inflammation and the maturation of B cells	3.59
CXCL2	X53799	Chemokine with inflammatory activity	3.23
IL-4	M13982	Pleiotropic cytokine, regulator of NO synthesis	2.62
CCL-2 or (MCP-1)	M24545	Chemokine activity, immunoregulatory and inflammatory processes	2.31
TGF-β	X02812	Embryogenesis and cell differentiation	2.01
IL-3	M14743	Cell growth, differentiation, apoptosis	0.47
Pleiotrophin	M57399	Growth factor, heparin binding activity	0.25
**Receptors**			
CCR-2	U03905	Monocyte infiltration, in inflammatory diseases	10.40
IL-10Rα	U00672	Inhibits the synthesis of proinflammatory cytokines, promote survival of progenitor myeloid cells	8.63
IL-12Rβ	U03187	Receptor for IL-12	6.17
TRAILR-1	U90875	Transduces cell death signal and induces cell apoptosis	4.91
IL-9R	M84747	The ligand binding of this receptor leads to the activation of various JAK kinases and STAT proteins	4.71
CD40	L07414	T cell-dependent immunoglobulin class switching, memory B cell development	4.58
Fas	M67454	physiological regulation of programmed cell death	4.21
IL-6R	X12830	Regulates cell growth and differentiation and plays an important role in the immune response	4.16
IL4R	X52425	Interleukins & Interferons receptors	3.91
IL-8Rα	M68932	Interleukins & Interferons receptors	3.33
IL-2Rα or CD25	X01057	Transduction of mitogenic signals from IL-2	3.00
IL-2Rγ	D11086	Signaling initiation and T cell proliferation	2.38
Complement component 5 receptor 1	M62505	Defense response to bacteria	0.45
IL-3Rα	M74782	Interleukins & Interferons receptors	0.43
**Others**			
S100 calcium binding protein A8 (calgranulin A)	X06234	Cell cycle progression and differentiation	7.18
Interferon regulatory factor 8 (IRF-8)	M91196	Transcription Activators & Repressors	6.16
S100 calcium binding protein A9 (calgranulin B)	X06233	Cell cycle progression and differentiation	4.28
PTK7 protein tyrosine kinase 7	U33635	Cell adhesion molecule, intracellular transducers, effectors & modulators	3.92
IRF-1	X14454	Transcription activator of genes induced by IFN-α, β, γ and apoptosis	3.09
TEK tyrosine kinase, endothelial	L06139	TEK signaling pathway critical for endothelial cell-smooth muscle cell communication	2.78
AXL receptor tyrosine kinase	M76125	Stimulation of cell proliferation and cell aggregation	2.53
RYK receptor-like tyrosine kinase	S59184	Intracellular transducers, effectors & modulators	2.29
Thrombomodulin	M16552	Thrombin binding, activation of protein C,	0.42
PDGFA associated protein 1	U41745	Fibroblast growth	0.31
Thrombopoietin	L36052	Megakaryocyte proliferation, maturation and thrombopoiesis	0.23

### Validation of gene expression by Real time-PCR confirmed array results

**Figure 1:**
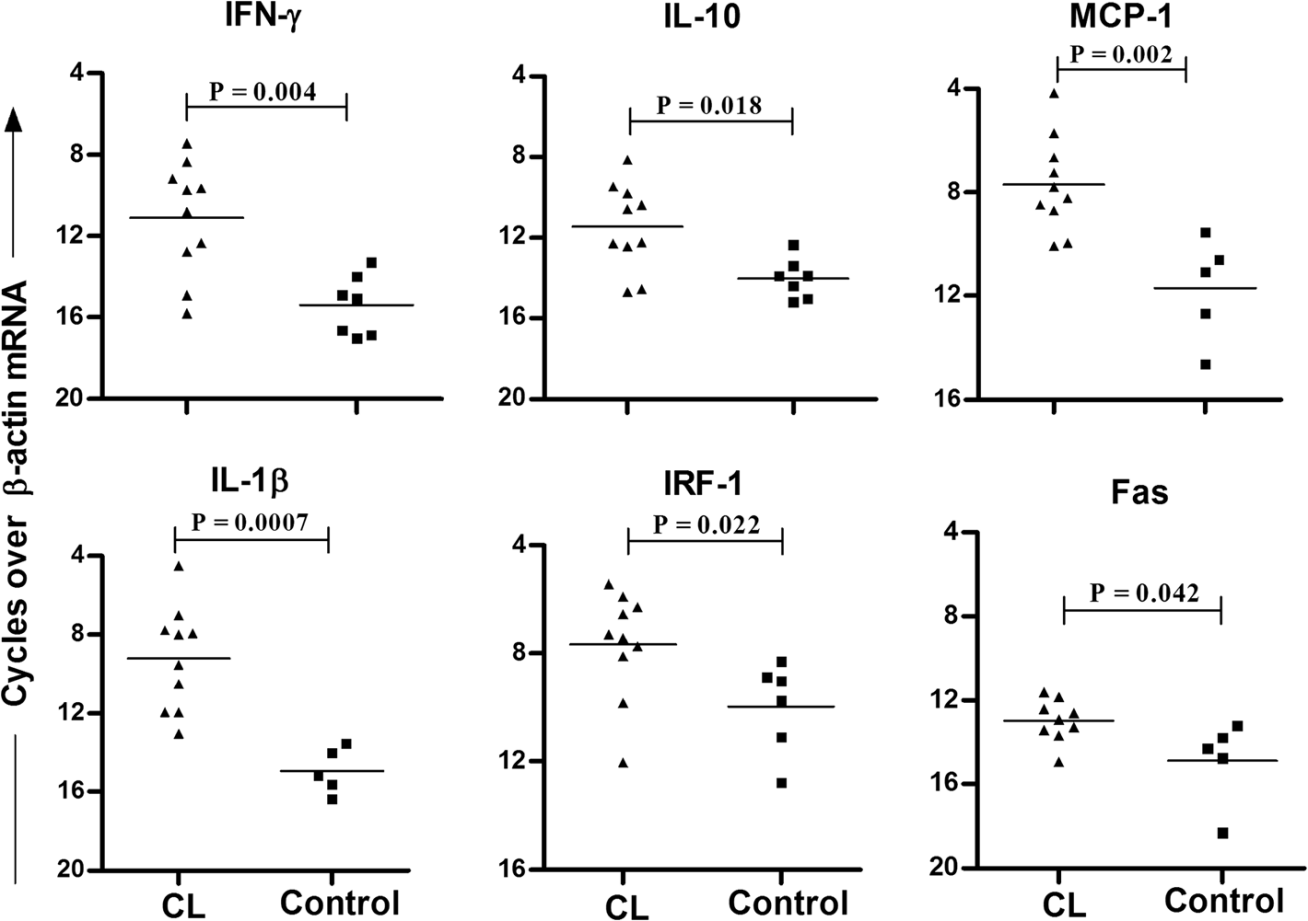
**Validation of cDNA array results using real time PCR in tissue lesions of CL patients.** Relative mRNA levels of IFN-γ, IL-10, MCP-1, IL-1β, IRF-1 and Fas were determined by QPCR in tissues lesions of CL patients (n = 10) or control tissues (n = 7). The relative quantification of products was determined by the number of cycles over endogenous control (β-actin mRNA) required to detect the gene expression of interest. Values of *P* were calculated using unpaired Student’s t test. Horizontal lines indicate mean values.

Selected genes showing differential expression on cDNA array were validated in individual CL samples. The rationale of gene selection was to include representative genes from all three functional categories given in Table 2 viz. cytokine/chemokine (including pro-inflammatory, anti-inflammatory cytokines and a chemokine), receptors (Fas, important in apoptosis) and others (IRF-1, important for IL-12 production and Th1 responses). Within the cytokine/chemokine category we selected the genes reported to be important in CL pathogenesis. Analysis of mRNA by real-time PCR confirmed elevated mRNA for IFN-γ, IL-10, MCP-1, IL-1β, IRF-1, and Fas in pretreatment lesion tissues compared with control tissues. Out of 7 control samples, mRNA level of MCP-1 and IL-1β was not detectable in two, while IRF-1 level was not detectable in one sample (Figure 1).

### Active CL is associated with elevated mRNA levels of Th-17 and Treg cells markers in lesion tissues

Elevated expression of IL-17 and FoxP3 mRNA is reported in CL patients with *L. major* as the causative species [11,13]. We have recently demonstrated elevated levels of IL-17 and Foxp3 in lesion tissue of PKDL patients, and a direct correlation between Foxp3 mRNA levels with parasite load [15]. In the present study we noticed elevated expression levels of IL-17 and CD25 on cDNA array that provoked us to investigate in detail the involvement of Th17 and Treg cells in CL lesion tissue and its association with disease pathogenesis.

**Figure 2:**
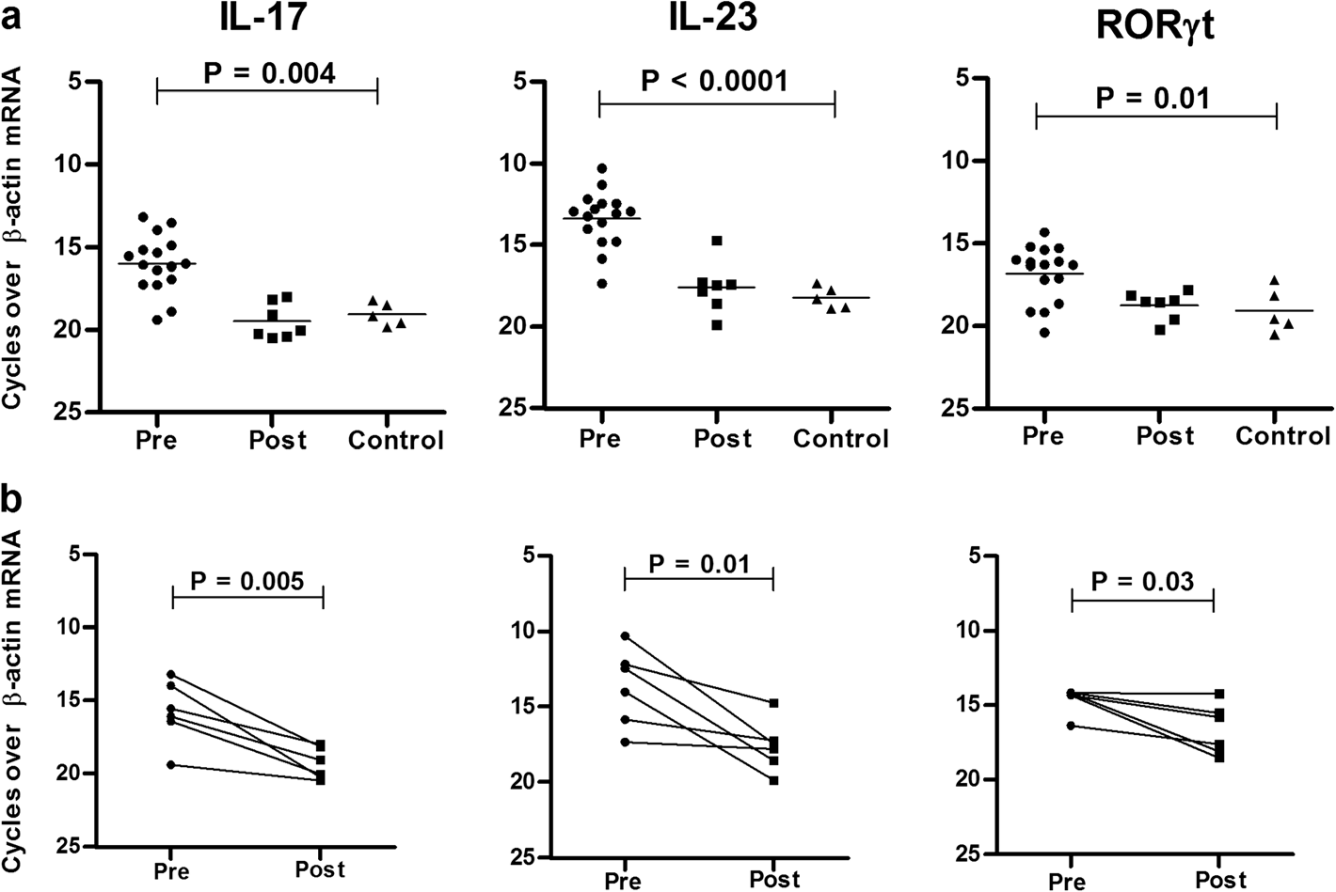
**mRNA expression of Th17 markers in CL.** Relative mRNA levels of IL-17, IL-23 and RORγt was determined by QPCR in tissues lesions at **(a)** pretreatment (n = 16), post treatment (n =7), and control tissues (n = 5), **(b)** in paired samples (n = 6). The mRNA level represents the number of cycles over endogenous control (β-actin) required to detect the gene expression of interest. Values of *P* were calculated using unpaired Student’s t test for unpaired samples in panel **(a)** and paired t test for paired samples in panel **(b)**. Horizontal lines indicate mean values.

Analysis of mRNA by real-time PCR confirmed significantly elevated levels for Th17 markers (IL-17, IL-23 and RORγt) and Treg markers (CD25, Foxp3 and CTLA-4) in tissue lesions of CL (n = 16) compared with healthy controls (n = 5). (IL-17 *P* = 0.004; IL-23, *P* < 0.0001; RORγt, *P* = 0.01) (Figure 2a). In post-treated cases (n = 6), significantly reduced mRNA levels of IL-17(*P* = 0.005), IL-23 (P = 0.01) and RORγt, *P* = 0.03) were evident in paired samples (Figure 2b).

**Figure 3:**
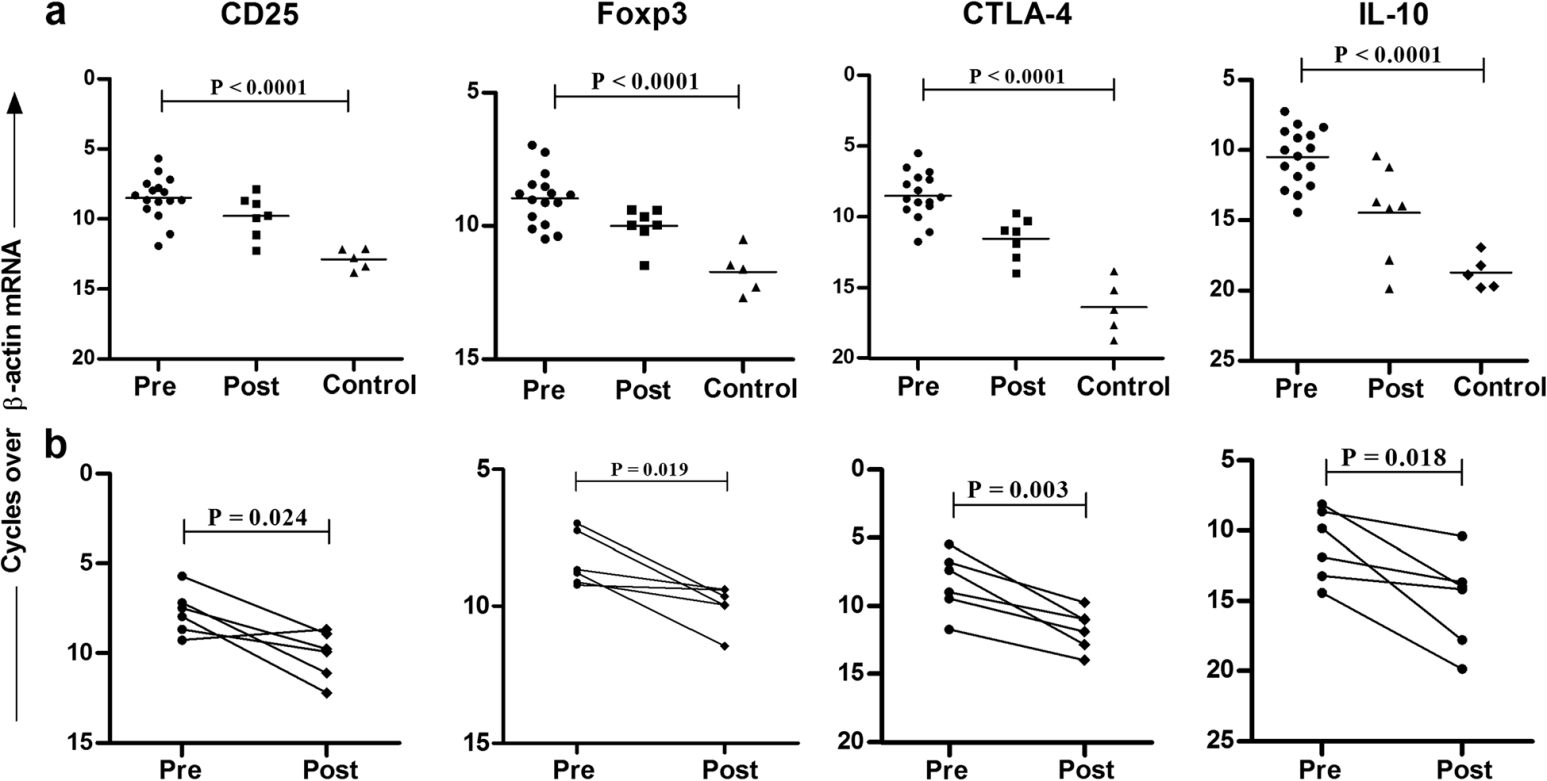
**mRNA expression of Treg markers in CL.** Relative mRNA levels of CD25, CTLA-4, Foxp3 and IL-10 was determined by QPCR in tissues lesions at **(a)** pretreatment (n = 16), post treatment (n =7), and control tissues (n = 5), **(b)** in paired samples (n = 6). Values of *P* were calculated using unpaired Student’s t test for unpaired samples in panel **(a)** and paired t test for paired samples in panel **(b)**. Horizontal lines indicate mean values.

CD4^+^CD25^+^Foxp3^+^ regulatory T (nTreg) has inhibitory effects on mouse Th17 lineage development, and Treg are associated with drug unresponsiveness and disease pathology in human CL [13,14]. Analysis of mRNA levels of nTreg markers CD25, CTLA-4 and Foxp3 revealed elevated levels of nTreg markers in pre-treatment cases compared to control (*P* < 0.0001) (Figure 3a). After treatment, a significant decrease in mRNA levels (CD25, *P* = 0.024; Foxp3, *P* = 0.019; and CTLA-4, *P* = 0.003) was noticed in paired samples (Figure 3b).

### Elevated IL-10 mRNA levels in lesion tissues of CL patients

Several IL-10–producing CD4^+^ T cell sub-populations, B cells and professional APC has been frequently associated with the suppression of anti leishmanial immune responses in human experimental models. mRNA analysis in skin tissues revealed significantly elevated IL-10 mRNA levels in localized tissues of CL patients compared to control (*P* < 0.0001) (Figure 3a). Further, IL-10 transcripts was found significantly elevated in pre-treatment compared with post-treatment samples (*P* = 0.018) (Figure 3b).

### Active CL is associated with elevated levels of IL-17 in plasma

**Figure 4:**
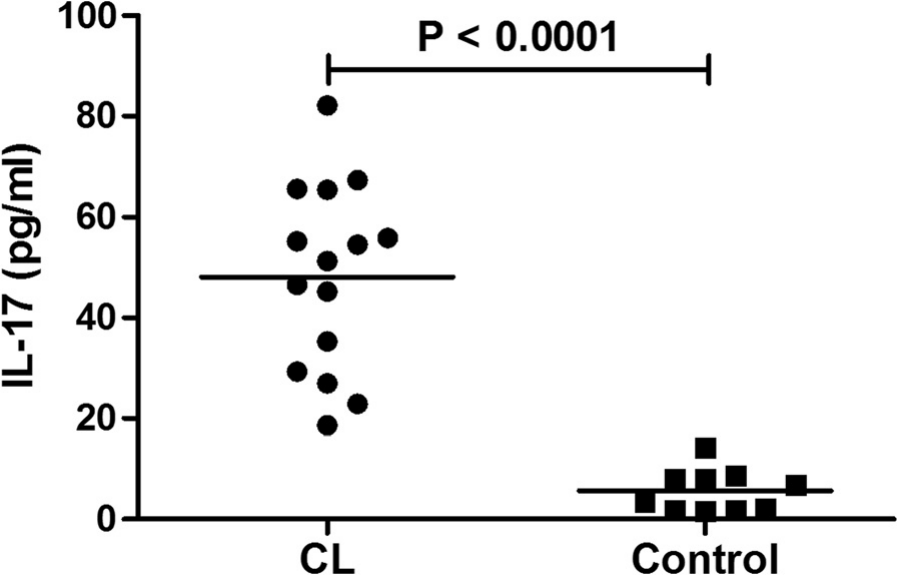
**Plasma levels of IL-17 in CL.** Plasma levels of IL-17 was determined by ELISA in CL patients (n = 15) and controls (n = 10). Individual values (pg/ml) are presented and the horizontal lines indicate mean values. Values of *P* were calculated using the Mann–Whitney test.

To corroborate the localized mRNA level with protein level, circulating IL-17 levels was analyzed in plasma samples of CL patients at pretreatment (n = 15) and healthy controls (n = 10) using cytokine ELISA. Levels (pg/ml) of IL-17 was found to be significantly higher in CL samples compared to control (mean ± SE, 48.14 ± 4.769; 5.69 ± 1.32) (*P* < 0.0001) (Figure 4).

## Discussion

Most studies on human leishmaniasis use cells from the peripheral blood, which may not be a representative image of the specific immune response at the site of the infected lesion tissue. Here we utilized a nylon membrane cDNA array to capture localized immune responses in lesion tissues of CL patients. The present study beyond doubt provided vital information about array of immune modulators (cytokines/chemokines /receptors /apoptotic /regulatory molecules) that are differentially modulated in lesion tissues of host upon infection with *L. tropica.*

Previously, cDNA array technology has been successfully used to analyze transcription in host cells in response to several intracellular pathogens including *Leishmania*[16-18], *Salmonella*[19], and *Chlamydia*[20]. These studies were performed in cell lines or experimental models and studies are still lacking to address specifically the questions associated with respect to *L. tropica* infection in humans. In experimental leishmaniasis, the activation of macrophage and production of Th1 cytokines, IFN-γ and TNF-α, are required to eliminate *Leishmania* parasite [21]. Our data shows elevated levels of IFN-γ and TNF-α in lesion tissue of CL patients. In our earlier studies in human CL and PKDL, we have documented elevated levels of intralesional transcripts of IFN-γ and TNF-α along with IL-4 and IL-10 [8,15,22]. Thus, the present study indicates that in spite of presence of effector molecules in *L. tropica* infected dermal tissues; the lesions persist possibly due to simultaneous presence of immunosuppressive molecule IL-4 and IL-10.

Our cDNA array data revealed highly elevated mRNA levels of IL-12β (IL-12p40) in lesion tissues of CL patients, similar to findings in PKDL patients [23]. IL-12 is necessary for the development of a Th1 response. IL-12p40 subunit is shared by IL-12 and IL-23 cytokines, giving the possibility for bioactive IL-12p70 or IL-23 or suppressive IL-12p80. IL-23, a heterodimeric, IL-12 family cytokine member is required for differentiation and maintenance for Th17 cells [24]. The cDNA array results demonstrated elevated IL-17 mRNA levels in lesion tissues of CL patients, indicating involvement of Th17 type responses in the disease biology. Based on these results, further investigation on Th17 markers in individual patients revealed elevated mRNA levels of IL-17, IL-23p19 and RORγt at pre-treatment stage. Further, the message IL-17 data was in corroboration with the protein data. The role of Th-17 cells in leishmaniasis is not clear. In the case of CL and ML it is associated with tissue pathology, whereas in VL it is associated with protection [10,11]. However, another study from India has demonstrated no difference in protein or message levels of IL-17and RORγt between the pre- and post-treatment groups in VL. Furthermore, the study showed significantly reduced levels of IL-23p19 in VL, a molecule required for differentiation and maintenance for Th17 cells [25]. In contrast, our recent study demonstrated the role of Th17 responses in PKDL pathogenesis [23]. Since PKDL, is a sequel to VL, reasons for possible discrepancy between VL, PKDL and CL could be (i) in VL, the infection is systemic whereas both PKDL and CL are dermal infections; (ii) Th17 responses are more profound in skin [11,23,24,26]; (iii) VL lacks *Leishmania* specific T cell responses whereas both PKDL and CL show T cell proliferation against *Leishmania* antigens.

Furthermore, severe cutaneous pathology was also associated with *L. major* infection in IL-27Ra–deficient mice, which was associated with striking increase in Ag-specific IL-17-producing CD4^+^ T cells [26]. Because IL-27 has inhibitory effects on mouse Th17 lineage development, it will be of interest to investigate the effect of IL-27 on IL-17 pathway in Indian CL patients. IL-17 is associated with inflammation and recruitment of neutrophils at the infection site during *L. major* infection in humans [11,27]. Also IL-8 is a strong neutrophil chemotactic factor. In our array results, both IL-17 and IL-8 showed substantial elevated expression, which is in agreement with the histochemistry report demonstrating inflammatory infiltrate predominantly of neutrophils (MPO staining) and strong IL-8 expression in lesion tissues of CL patients [7].

Histopathologic analysis of lesion tissues of CL patients show mixed granulomas consisting of macrophages, lymphocytes, epitheloid, and plasma cells; and chemokines such as CXCL-9, MCP-1, MIP-1α and MIP-1β are the potent chemoattractant of T cells, monocyte and macrophages. Previously, we have reported elevated levels of circulating and localized MCP-1 in lesion tissues of CL [7]. Here we are reporting elevated expression of these chemokines in lesion tissues of CL patient. Further, in experimental model *L. tropica* infected strains exhibited increased levels of chemokines CCL2, CCL3 and CCL5 [28]. These observations reflect association of these chemokines in host pathogen interaction and their role in the disease biology.

Beside Th1, Th2 and Th17 cell subsets, CD4^+^ T cells also include CD25^+^ Foxp3^+^ T regulatory cells. Numerous recent observations have demonstrated that functional immunity to several microbes is influenced by Tregs. At the pre-treatment stage the expression of Treg markers (CD25, CTLA-4 and Foxp3) along with IL-10 was found significantly elevated in CL tissue lesions compared to control, implicating a crucial role of Tregs in *L. tropica* infection. Earlier studies on human leishmaniasis have demonstrated involvement of intra-lesional Tregs in local control of effector T cell functions and correlation with drug unresponsiveness and parasite load [13-15].

The present study demonstrated the coexistence of a two antagonistic T cell responses i.e. proinflammatory (Th17) and regulatory (Tregs) response in Indian CL caused by *L. tropica*. A number of studies have identified co-existence of Th17 and Treg cells in various diseases [29-31]. Abundance of inflammatory cytokines (TNF-α, IL-1β and IL-17) in CL supports the extensive inflammatiory nature of the disease which causes tissue damage at the site of infection. The presence of Tregs in infected tissues may be a possible homeostatic mechanism to control infection-induced inflammation. However, it requires further investigation to clarify whether the accumulation of Tregs is directly induced by parasite or it is a homeostatic process of host immune system to suppress excessive inflammatory responses directly or indirectly, supporting parasite survival through IL-10 mediated immune suppression.

## Conclusions

The study provided a comprehensive picture of immune-determinants inside lesion tissues of CL patients, indicating a role of Th17 and Treg cells in CL caused by *L. tropica*. The snap of immunological scenario in the present study beyond doubt provides vital information related to the ongoing pathological process in localized lesion tissues of CL patients, relevant from point of developing strategies for control and prevention of the disease.

## Abbreviations

CL: Cutaneous Leishmaniasis; ML: Mucosal Leishmaniasis; VL: Visceral Leishmaniasis; PKDL: Post Kala-Azar Dermal Leishmaniasis; IL: Interleukin; IFN: Interferon; Th: T Helper cell; TNF: Tumour Necrosis Factor; MCP: Monocyte Chemoattractant Protein.

## Competing interests

The authors declare that they have no competing interests.

## Authors’ contributions

PS, GKK, AR and RK conceived and designed the experiments. GKK, AR, KA and HK performed the experiments. PS, GKK, NAA and RK analyzed the data. PS and RAB Contributed reagents/materials/analysis tools. PS, GKK, NAA and RK wrote the paper. All authors read and approved the final manuscript.

## Authors’ information

GKK, PhD, AR, MSc, RK, PhD, KA, MSc, HK, MSc, NAA, PhD, RAB, MD, PS, PhD.
